# Continuing Shifts in Epidemiology and Antifungal Susceptibility Highlight the Need for Improved Disease Management of Invasive Candidiasis

**DOI:** 10.3390/microorganisms10061208

**Published:** 2022-06-13

**Authors:** Ben Y. Parslow, Christopher R. Thornton

**Affiliations:** 1 Biosciences, College of Life and Environmental Sciences, Geoffrey Pope Building, University of Exeter, Stocker Road, Exeter EX4 4QD, UK; bp342@exeter.ac.uk; 2 Medical Research Council Centre for Medical Mycology, Geoffrey Pope Building, University of Exeter, Stocker Road, Exeter EX4 4QD, UK

**Keywords:** *Candida*, invasive candidiasis, candidemia, epidemiology, antifungal, diagnostics

## Abstract

Invasive candidiasis (IC) is a systemic life-threatening infection of immunocompromised humans, but remains a relatively neglected disease among public health authorities. Ongoing assessments of disease epidemiology are needed to identify and map trends of importance that may necessitate improvements in disease management and patient care. Well-established incidence increases, largely due to expanding populations of patients with pre-disposing risk factors, has led to increased clinical use and pressures on antifungal drugs. This has been exacerbated by a lack of fast, accurate diagnostics that have led treatment guidelines to often recommend preventative strategies in the absence of proven infection, resulting in unnecessary antifungal use in many instances. The consequences of this are multifactorial, but a contribution to emerging drug resistance is of primary concern, with high levels of antifungal use heavily implicated in global shifts to more resistant *Candida* strains. Preserving and expanding the utility and number of antifungals should therefore be of the highest priority. This may be achievable through the development and use of biomarker tests, bringing about a new era in improved antifungal stewardship, as well as novel antifungals that offer favorable profiles by targeting *Candida* pathogenesis mechanisms over cell viability.

## 1. Introduction

Until relatively recently, fungi were a rare cause of life-threatening human disease. Since the early 1980s, invasive fungal diseases (IFDs) have been an increasing occurrence in healthcare environments due to an ever-expanding population susceptible to infection [1,2]. This has largely been driven by the advent of more aggressive interventions and treatments in modern healthcare, placing patients in prolonged states of severe immunosuppression [3,4].

**Figure 1:**
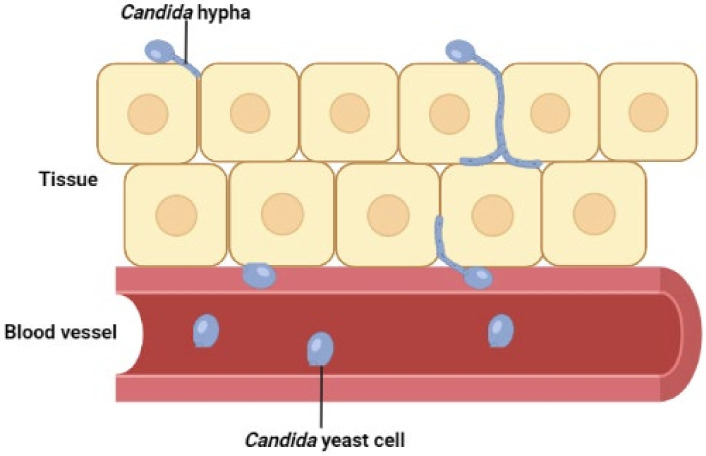
Invasive candidiasis involves rapid dissemination of *Candida* yeast cells in the bloodstream (candidemia BSI) and/or tissue penetration by invasive hypha (deep-seated candidiasis). Hematogenous seeding of *Candida* yeast from blood-borne candidemia is often a key source of tissue candidiasis and vice versa [9]. Although hyphal extension is a well-described mechanism of *C. albicans* tissue invasion, its role is less clear for other clinically important *Candida* species such as *C. glabrata* where other mechanisms of invasion may be involved.

Invasive candidiasis (IC), caused by yeast species in the fungal genus *Candida*, is one of the main systemic, opportunistic fungal diseases of immunocompromised patients. It is associated with significant global burden, with an estimated 750,000 cases occurring annually [5] and unacceptably high mortality rates of up to 30% [6,7]. More than 15 *Candida* species have now been described as etiologic agents of IC, but >90% of cases are attributed to just five: *C. albicans, C. glabrata, C. parapsilosis, C. tropicalis and C. krusei.* Of these, *C. albicans* is predominant [1,8]. The term invasive candidiasis is used to describe two distinct disease entities: candidemia bloodstream infection (BSI) and deep-seated tissue candidiasis (Figure 1), which may occur independently or concomitantly [9].

Despite being an increasing cause of morbidity and mortality in healthcare settings, IC is still a somewhat neglected topic among public health authorities. Consequently, improvements in disease management and patient care are urgently needed [1,5]. Of particular importance is a lack of fast, accurate diagnostics, with clinicians continuing to rely on suggestive clinical findings and the use of culture-based diagnostics with sub-optimal sensitivity to inform decision making [10]. It is suggested that up to 50% of disease episodes may go undiagnosed by these conventional methods, resulting in delayed treatment initiation and markedly worse patient outcomes [9,10,11]. To mitigate this, current treatment guidelines recommend that prophylactic or empirical preventative strategies be initiated in high-risk populations in the absence of proven infection [12,13]. Whilst some patients will benefit from these measures, non-specific implementation leads to unnecessary use of precious antifungals in many instances. This has led many to raise concerns about the risks of widespread drug resistance among *Candida* spp., particularly given the already limited availability of front-line antifungals [14,15].

Overall, IC represents a major global public health concern. A robust understanding of ongoing disease epidemiology is therefore of importance to identify concerning trends that may inform policy decisions and necessitate the need for improvements in disease management and patient care. In this review, a critical appraisal of the current IC epidemiologic landscape is made, focusing on prospective surveillance studies that assess patient pre-disposing risk factors, incidence, *Candida* spp. distribution and antifungal susceptibility patterns both spatially and temporally. Particular attention will be given to the interplay between these factors. Antifungal treatment and diagnostics are outlined as two key components of IC management, with the influence of current practices on disease epidemiology considered. Viable improvements in the implementation of these clinical activities are then proposed, offering the potential for a new era in disease management and patient care through improved antifungal stewardship and availability.

## 2. Pre-Disposing Risk Factors

As a commensal fungus that is part of the normal human microbiota, *Candida* very rarely causes invasive infections in healthy individuals [16]. Classically, life-threatening IC infections are found in patients with compromised immune systems [3,17,18,19], broadly influenced by healthcare and host-related risk factors [4,20].

### 2.1. Healthcare Factors

Healthcare-related pre-disposing risks for IC often involve cutaneous or mucosal barrier disruption and broad-spectrum antimicrobials, common interventions in healthcare. Cutaneous barrier disruption is often a result of subcutaneous medical devices, such as central venous catheters (CVCs) and portacaths, implanted for long-term intravenous drug infusion or parenteral nutrition [4]. Subcutaneous implanted medical devices into the vasculature are a common source of candidemia BSI. This is due to the ability of *Candida* to form biofilms on such indwelling abiotic surfaces and subsequent release of *Candida* yeast cells into circulation [16].

Disruption of the mucosal barrier in the gastrointestinal tract through surgery or transplantation is also an important risk factor for invasive infection, resulting in increased translocation of gut-dwelling *Candida* into the vasculature [21]. Administration of broad-spectrum antimicrobials increases colonisation and pathogenic capabilities of *Candida* spp. by altering the interactions and dynamics of the microbiome. A distinct shift is seen from mutualism and colonisation in commensal *Candida* to competition and infection in IC [22,23]. Others postulate that chemotherapy agents may have similar effects on the gut microbiome [24]. Together with their immunosuppressive activity, chemotherapeutic agents represent a major risk factor by acting through multiple channels.

### 2.2. Host Factors

Underlying host conditions can bring about immunocompromised states that increase the likelihood of opportunistic infection. This may occur directly, such as in HIV infection, some cancers, renal disease, liver cirrhosis and diabetes mellitus among others [4,25,26], or indirectly through immunosuppressive treatment (e.g., chemotherapy, corticosteroids and, more recently, immunotherapy) for underlying conditions such as solid and haematological malignancies [3]. These populations of the immunosuppressed have expanded in recent decades with the advent of longer treatment regimens and newer therapeutics with greater cytotoxic payloads and potency [2,27]. Of particular importance is the increasing number of neutropenic individuals, defined as a neutrophil blood count <0.5 × 10^9^/L, who are extremely vulnerable to infection due to their inability to mount an effective immune response to invasive *Candida* [24,28]. Despite this, others have found a lack of correlation between immunocompromised individuals and IC prevalence [29,30,31]. Considered in isolation, the state of being immunocompromised may be a small contributor to IC risk, but the simultaneous presence of other healthcare risk factors, acting as an initial source of systemic *Candida*, may act to increase the relative risk of immunosuppression. This may explain the disparity in studies on pre-disposing risk factors and outlines the difficulty in defining the relative risk posed by one individual factor when, clinically, interplay exists, with multiple risk factors often observed together [4,31]. Therefore, a holistic approach may be more appropriate, taking account of all major risk factors in combination.

## 3. Incidence

Globally, IC incidence has generally increased over the last decades as the populations susceptible to infection have expanded [2,32,33], with increases in both hospital- and community-acquired infections observed. *Candida* is now responsible for ~10% of nosocomial BSIs in the United States (US) [1,2,34,35], and up to one-third of episodes are also thought to be community-acquired due to the increasing at-home management of patients with CVCs [8]. Despite a well-described incidence increase, recent studies suggest that this may be stabilising or reversing in certain settings [36,37,38,39,40]. The reasons for these observations are not well-defined, but improvements in the management of patient pre-disposing risk factors [36,37], and increasing uptake in biomarker and prediction tool use (e.g., T2 *Candida*, Colonisation Index and Acute Physiology and Chronic Health Evaluation II score) that allow for targeted preventative treatment strategies in identified high-risk patients have been suggested [41]. Importantly, to date, studies reporting this decline are limited, with a greater number of data sets required to establish these observations as an ongoing trend more widely. Specifically, the importance of improved risk factor management, as well as biomarker and prediction tool use that have already been suggested in these declines, should be investigated further. If a causal relationship is widely established, this should act to drive broad changes in IC disease management strategies that implement these healthcare activities.

When assessing IC incidence, several limitations must be acknowledged. First is the use of different denominators in incidence calculations, making it challenging to draw meaningful comparisons. Population-based studies report incidence as cases per 100,000 persons, whilst hospital-centre-based studies often report it as cases per 10,000 patient days or 1000 admissions [42]. Comparisons of incidence are also challenged by the variable sensitivity of blood culture, influenced by numerous factors that are not standardised across healthcare settings. These include the blood culture system in use, blood culture volume and whether antifungals were in use at the time of blood draw [36,43]. Of primary importance is that most surveillance studies are inherently flawed. This is because they look only at a partial disease spectrum, focusing on candidemia BSI and often disregarding deep-seated tissue candidiasis. This is due to the current difficulties in diagnosing deep-seated infection, meaning these disease events are largely under-ascertained [9,10]. Furthermore, suboptimal sensitivity of current diagnostics for IC infection generally means that candidemia BSI cases are also underdetermined, albeit to a lesser extent.

Inaccuracies and underestimation in IC cases and incidences are inevitable as estimates are based upon a partial clinical disease spectrum, diagnostics with poor sensitivity and factors that are not standardised across healthcare settings. Acknowledging these limitations, here an assessment of disease incidence will focus on prospective population-based surveillance data from the US and Europe, allowing for comparisons at the entire population level across the whole spectrum of clinical settings [44].

### 3.1. United States

Current estimates point to a higher disease incidence in the US than Europe [6,36,39,40,45], but since data are scarce and estimates vary greatly on a centre-to-centre basis such conclusions may not be nationally representative [6]. US data also suggest that whilst incidence has increased over the past decades, there may now be signs of reversal [6,36].

A series of studies aiming to provide accurate epidemiology data by conducting ongoing prospective population-based surveillance for candidemia BSI in metropolitan regions of four US states have been undertaken, with data available from 1992 to 2016 (Table 1) [6,36,44,46,47].

Incidence estimates varied greatly across the four sites during the surveillance period. In Georgia, incidence increased from 9.1 to 13.3 from 1992 to 2011 [44,46] before declining to 7.5 in 2016, respectively [6,36]. Similarly, in Maryland, incidence increased from 1998–2011, albeit to a lesser extent from 24.2 to 26.2 before declining significantly to approximately 13.0 in 2016 [6,44,47]. Data from Oregon and Tennessee are only available from a later date as these two sites were added to the CDC’s Emerging Infections Program [EIP] for candidemia surveillance in 2011. In Oregon, incidence remained relatively stable and significantly lower than other sites at 3.0–3.5 whilst in Tennessee incidence rose markedly in a short 4-year period by 4.5 cases per 100,000 [6].

**Figure 2:**
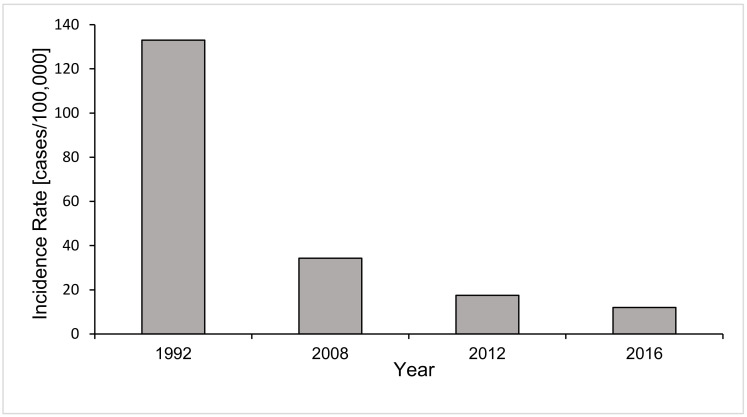
Annual candidemia incidence rates in infants aged ≤1 year described from surveillance at a metropolitan site in Georgia in 1992, 2008, 2012 and 2016 [6,44,46]. Candidemia incidence in this age demographic shows a declining trend, with incidence decreasing markedly from 133.0 to 12.0 cases per 100,000 population in 1992 and 2016, respectively.

The main drivers of both increasing and decreasing incidences were identified. Adults aged >65 in ICUs were of greatest importance in observed increases, with this demographic now representing the largest contribution to total cases with an incidence of 25.5 per 100,000 persons [6,44,46,47]. Increasing numbers of total patients >65 in ICUs may be implicated, where invasive procedures that put elderly patients at risk for opportunistic IC infection are particularly common. IC was previously known to be most prevalent among those aged ≤1 year, driven by low-birthweight and pre-term infants. Although this age demographic still represents an important contributor to IC caseload, drastic declines in incidence have been observed (Figure 2) [6,44,48].

Low-birthweight and pre-term infants aged ≤1 year often require implanted medical devices, such as CVCs to be fitted, an important pre-disposing risk factor for IC infection. Therefore, improvements in infection control practices around hand hygiene and maintenance of these devices have been implicated in this trend [44,48]. Widespread implementation of standard-of-care antifungal prophylaxis may also be contributory [48]. In addition to age-specific declines, incidence decreases have been described overall from 2011 at sites in Georgia and Maryland [36]. Again, improvements in the management of patient risk factors are thought to be important, with CVC-associated candidemia almost halving from 2008 to 2013 whilst cases in patients without CVCs remained constant [36]. In the US, increasing incidence therefore appears to have been driven largely by the >65 age demographic in ICU whilst recent incidence declines result from improved management of patient pre-disposing risk factors, particularly among infants.

State- and site-specific incidences vary considerably, with the influence of varying risk factor prevalence in underlying populations and implementation of disease management strategies considered. It was found that 77% and 73% of culture positive candidemia cases were associated with systemic antimicrobial administration and patients with CVCs, respectively [6], outlining the major infection risk that they pose. Differences in the patterns of use of these healthcare factors will therefore influence the population susceptible to infection and subsequent IC incidence. The impact of underlying conditions such as solid/haematological malignancies that require prolonged immunosuppressive treatment is apparently negligible given that these patients account for a small proportion of the total population susceptible to IC (~5% of total cases only [1,6,49]). Varying implementation of preventative treatment and other disease management strategies that might reduce group-specific incidence in these high-risk populations may be important [36,37,41,50]. Surprisingly, 10% of disease events were associated with patients that had a history of injection drug use (IDU), a risk factor previously considered to be of less prominence. It is hypothesised that the current opioid crisis in the US and associated increased IDU rates might be responsible [51,52]. Importantly, while several factors are outlined here, defining the impact that any one factor has on incidence of IC is problematic. Furthermore, it must be acknowledged that there will be many other contributory factors that are currently not well-defined.

### 3.2. Europe

Since the first population-based European incidence data were described in 1980 [53], estimates have ranged from 1.4 to 10.05 cases per 100,000 population [39,40,45,53,54,55,56,57,58,59,60], outlining the considerable temporal and spatial variation that exists. Despite this large variation, available estimates to date are generally lower than those described in the US.

As two of the few countries that have ongoing national prospective surveillance for candidemia, Iceland and Denmark will be examined here to assess potential trends and enable comparisons with US data. Incidence in Iceland increased steadily from 1980 to 2009, where it peaked at 7.5 cases/100,000 [39,53]. In the subsequent two years, incidence declined markedly to 4.0 cases/100,000 persons, consistent with trends observed in the US and other European countries [6,36,39,40]. In these countries, declines were attributed to improved management of patient pre-disposing risk factors. However, in Iceland, it is noted that the volume of blood cultures performed at this time also reduced considerably due to an ongoing economic recession, and that this may invalidate these findings somewhat. Further readouts of incidence from Iceland are therefore required to accurately assess whether declines have truly occurred [39].

Denmark represents an outlier in European IC incidence estimates which, through its national surveillance programme for candidemia BSI, has consistently reported some of the highest regional incidences over 15 years [40,59,60], peaking at 10.05 cases/100,000 persons in 2011 [60]. The reasons suggested for this are two-fold. Danish surveillance may be more accurately ascertaining disease episodes, or the true disease burden may be greater with increased/different antibacterial drug use suggested as a cause for this [54]. Specifically, one Danish study observed that IC risk was disproportionately increased in critically ill ICU patients exposed to ciprofloxacin containing antibiotics compared with other antibacterial regimens such as cefuroxime and piperacillin [61]. Differences in national IC disease burdens will exist but are unlikely to account entirely for the significantly higher incidence in Denmark compared to neighbouring countries. Therefore, the more important factor might be the presence of robust national surveillance for candidemia that yields a more accurate ascertainment of total IC caseload across Danish healthcare than elsewhere. This highlights the importance of robust national surveillance systems for IC in understanding disease epidemiology and may be used as an appropriate model for other countries and regions to follow.

## 4. Antifungal Treatment

Antifungal agents (Table 2) are limited because their development relies on the identification of fungus-specific targets, which is challenged by the phylogenetic similarities between the fungal and animal kingdoms, both being eukaryotes [15]. As a life-threatening invasive mycosis, prompt initiation of intravenous antifungal treatment in suspected IC cases affects prognosis markedly [11]. Treatment guidelines therefore often recommend prophylactic and empirical strategies, due to a lack of fast, accurate diagnostics [12,13]. Prophylaxis involves preventative antifungal treatment for high-risk populations in the absence of infection signs and symptoms whilst empirical treatment is initiated in patients with suggestive clinical findings of infection in the absence of a clear diagnosis [13,62]. Given the implications of delayed treatment and the difficulties associated with making a fast and accurate diagnosis, these pre-emptive strategies have been the preferred means of managing patients at risk for IC with antifungal drugs.

### 4.1. Polyenes

Polyenes were the first mainstay antifungal class approved for IC treatment [63], acting as fungicidal agents by irreversibly binding ergosterol in the *Candida* cytoplasmic membrane and forming aqueous pores that lead to ion leakage and cell death [64]. Despite excellent efficacy against species of the *Candida* genus, severe dose-limiting toxicities, most notably nephrotoxicity, meant that amphotericin B deoxycholate was never fully exploited clinically [65,66]. Now, reformulated lipid-based preparations termed ‘AmBisome’ (liposomal amphotericin B) benefit from an improved therapeutic index and toxicity profile by altering distribution to organs, although significant toxicity is still persistent [67,68,69]. Furthermore, as efficacy is not compromised, Ambisome is used under specific indications, particularly on haematology and oncology wards [67,70].

### 4.2. Triazoles

Triazoles represent the largest group of antifungals for IC treatment [15], displaying fungistatic activity by binding and inhibiting lanosterol-14α-demethylase, leading to ergosterol depletion in the *Candida* membrane [71]. As with polyenes, severe toxicities are well-documented, with hepatotoxicity often implicated in use. Additionally, a high risk for drug–drug interactions is of concern, which may adversely affect treatment responses if not managed correctly [62]. Despite this, triazoles still represent key therapeutic options due predominantly to limited alternatives, excellent efficacy and availability in both intravenous and oral preparations. For years, fluconazole was the primary treatment option for IC [12,14], but continuing emergence of resistance has now limited its clinical utility [12,13]. Drivers of resistance are not fully defined, but widespread clinical overuse of fluconazole is thought to be key, exerting strong selection pressures for *Candida* resistance [72]. The emergence of resistance means guidelines are increasingly recommending alternate drug classes for first-line therapy [12,13].

### 4.3. Echinocandins

Echinocandins are the newest antifungal class despite being introduced ~20 years ago, exhibiting important advantages over the different drug classes [2]. Owing to their fungus-specific target of β-D-glucan synthesis inhibition, they have markedly improved safety and toxicity profiles [73,74]. In addition, they exhibit few drug–drug interactions [13] and boast broad activity, remaining highly effective against *Candida* spp. displaying triazole tolerance. Importantly, *C. parapsilosis* and *C. guilliermondii*, due to a naturally occurring proline-to-alanine amino acid change in the Fks1 protein (Fks1p), are likely candidates for future echinocandin resistance and therefore should be monitored closely [75]. Fks1p represents the catalytic subunit of the β-1,3-glucan synthase complex, the target of echinocandins, meaning such amino acid polymorphisms in Fks1p may bestow resistance by evading echinocandin activity [76,77]. Superior toxicity profiles and minimal resistance to date mean that echinocandins are being increasingly favoured over triazoles and polyenes for IC treatment, except where *C. parapsilosis* is implicated in infection [12,13].

### 4.4. Antifungal Stewardship

Echinocandins represent a vital drug class in IC treatment. Few licensed drugs, unacceptable toxicities, triazole resistance and a relatively dry drug pipeline contribute to the scarcity of available alternatives [15]. The management of echinocandin use should therefore be of the highest priority. It is important that clinicians learn from mistakes made with fluconazole, aiming to reduce unnecessary echinocandin use where possible and limit emerging drug resistance [14]. A particular focus should be prophylactic antifungal practices in the absence of proven infection. More accurately, defining high-risk patients that might or might not benefit from such preventative treatment is highly desirable, and achievable through the development and use of biomarker tests that allow clinicians to accurately identify or rule out infection [9,10]. This could represent a new era, bringing improvements in disease management and patient care through improved antifungal stewardship. Given their potential importance, the applications and implications of different biomarker tests will be discussed in greater depth in Section 7.

## 5. Species Distribution and Antifungal Susceptibilities

Globally, more than 15 *Candida* spp. are known etiologic agents of IC [78,79], but the majority (>90%) of infections are attributed to just five: *C. albicans, C. glabrata, C. parapsilosis, C. tropicalis and C. krusei* [2,6,36,38,40,80]. As such, they will be the focus here. *Candida albicans* has long been and remains the predominant species present as clinical isolates from infected patients [1], but an ongoing global shift towards non-*albicans Candida* (NAC) species that exhibit decreased antifungal susceptibility is well-established [2,33,81]. Emerging multi-drug-resistant strains such as *C. auris*, first described in 2009, represent a serious public health threat and highlight further the concerning global divergence of *Candida* spp. implicated in IC to more resistant strains [82].

As with incidence, when assessing IC species distributions and antifungal susceptibilities, limitations in data must be considered. First is the sub-optimal sensitivity of blood culture, meaning data represent only the testing of isolates from disease events that were culture-positive [83], a partial spectrum of the total IC disease events. Furthermore, although undefined, inevitably not all culture positive cases will undergo species identification and antifungal susceptibility testing. However, as testing is common and even compulsory in some countries and healthcare settings [6,40], the volume of testing may be considered adequate and representative of the species distribution and antifungal susceptibility landscape. Comparisons are also challenged by factors that are not standardised across healthcare settings, with the use of different blood culture systems notable. For example, *C. glabrata* positivity rates have been shown to be higher where the BacT/Alert culture system is used [55,84]. Although this is clinically significant, quantification of differences between culture systems shows inconsistencies and will be impacted by other currently contested confounding factors [84,85]. As such, acceptable concordance will be assumed between blood culture systems in the isolation rates of *Candida* spp.

Here, an assessment of *Candida* spp. distribution and antifungal susceptibility will focus on spatial and temporal data shifts at the continental level. Additionally, specific consideration will be given to the role of patient pre-disposing risk factors in species distribution as well as the interaction between increased use of antifungals and shifts towards strains with less susceptibility [72].

### 5.1. Influence of Pre-Disposing Risk Factors

Pre-disposing risks for IC, both host and healthcare related factors, can influence the *Candida* spp. implicated in infection [6,72,86]. Increasing patient age, transplant procedures and prior fluconazole exposure are well-documented factors for increased isolation of *C. glabrata* [72,87,88,89,90]. The latter may point towards an applied selection pressure from fluconazole use, driving *C. glabrata*-implicated infection with greater drug resistance [91]. *C. parapsilosis* is renowned for its high prevalence among pre-term and low-birthweight infants [48,92,93,94]. This may be due to an elevated ability to form biofilms on indwelling devices, which are commonly used in this patient group [95,96]. Another key driver of *C. parapsilosis* infection is its ability for nosocomial spread, notably by hand carriage, leading to hospital outbreaks and persistence [94,97]. With the advent of at-home CVC management, *C. parapsilosis* may therefore also be responsible for the rise in community-acquired IC [8,98]. *C. tropicalis* and *C. krusei* have a heightened presence among severely neutropenic patients on oncology wards [99,100,101]. Oncology-specific *C. tropicalis* incidence now appears to be declining in certain settings, with widespread fluconazole prophylaxis and improved management of CVCs likely determinants in these observations [36,72]. Conversely, as with *C. glabrata*, fluconazole exposure is cited as a selection pressure that has acted in the emergence of resistant *C. krusei*, particularly among the oncology patient population [72]. However, reported increases in *C. krusei* prevalence pre-date widespread prophylaxis regimens [102], suggesting other factors have also influenced its emergence. These may include oncology-specific risk factors such as the long-term use of subcutaneous portacaths and CVCs as well as administration of certain chemotherapy agents [24].

### 5.2. Geographical Trends

**Figure 3:**
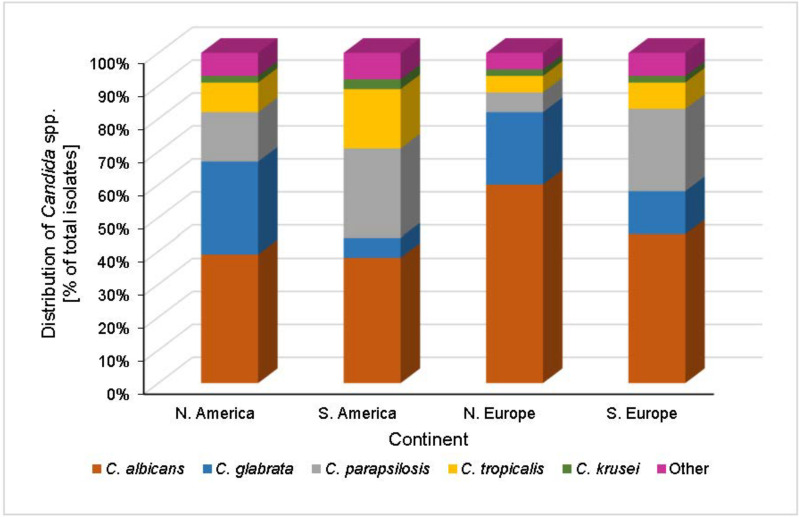
Distributions of *Candida* spp. as a percentage of total IC case counts across North America, South America, North Europe and South Europe [6,54,109,110,111]. In North America, *C. albicans* is the most prevalent *Candida* species, accounting for ~39% of IC episodes. The most common NAC species, representing just under 30% of cases, is *C. glabrata* with sequentially lower case contributions from *C. parapsilosis, C. tropicalis and C. krusei.* European IC species distribution shows two clear trends, split broadly between the north and south of the continent. Northern Europe typically exhibits a similar species distribution to North America, although a greater contribution from *C. albicans* is seen, accounting for 60% of cases. In contrast, the species landscape in southern Europe is more akin to that in South America, where *C. parapsilosis* is the most common NAC species. Across these four regions, <10% of total cases are attributed to species outside of the five described.

Species distribution and antifungal susceptibility shows considerable geographical variation between individual countries, but trends can generally be elucidated at the continental level, such as the Americas and Europe (Figure 3). Across some continents (Asia, Africa and Oceania), few distinctive trends are observed and are not well-defined due to limited and contrasting data from mostly single-institution studies [103,104,105,106,107,108]. As a result, data from Asia will be assessed briefly and Africa and Oceania will be excluded from this review.

#### 5.2.1. United States

*Candida glabrata* is of the highest concern in the US due to a combination of increasing incidence and high levels of resistance to front-line antifungals [6]. Up until the late 1990s, *C. albicans* accounted for ~50% of all *Candida* BSIs in the US [46], but its contribution has since decreased [6,36,44], with a concurrent increase in NAC incidence observed [1,6,36,44,46]. *C. glabrata* has emerged as the most frequent NAC species, making up 12% of isolates in 1999 [46] but now consistently accounting for just under 30% [6,36,44]. The significance of this trend is justified as *C. glabrata* exhibits high levels of triazole tolerance and emerging echinocandin resistance, albeit to a lesser extent [47,81]. Importantly, resistance shows considerable state variation perhaps due to differing patterns of population pre-disposing factors at a local level, outlining the importance of robust surveillance in local healthcare settings more widely. *C. glabrata* fluconazole resistance has been as high as 20% in selected US states (Georgia), but a gradual decline to ~10% has been observed since the 1990s [6,36]. Echinocandin resistance has increased simultaneously, with around 4% of *C. glabrata* isolates now displaying elevated MIC_90_ values (minimum concentration of antifungal required to inhibit growth of 90% of *Candida* cells) from susceptibility testing [6]. Decreasing clinical use of fluconazole in place of echinocandin as a first-choice treatment option is thought to be driving this shift, as selection pressures resulting from the use of these two drug classes change [44]. *Candida* echinocandin resistance remains low in most settings, but careful monitoring is required as clinical use inevitably increases [112]. *Candida parapsilosis*, *C. tropicalis* and *C. krusei* are much less frequently isolated, with events largely concentrated in specialist neonatal and oncology units. However, these NAC species also exhibit higher levels of antifungal resistance [91], suggesting the rapid emergence of *C. glabrata* in the US was mediated by several confounding risk factors in addition to selection for antifungal tolerant strains as the predominant factor [46]. It is noteworthy that *C. krusei* consistently exhibits fluconazole MIC_90_ values >64 μg/mL worldwide, rendering this antifungal of little use in *C. krusei*-implicated infections [44,46]. A more comprehensive national surveillance is required to track species-specific incidence and antifungal resistance trends.

#### 5.2.2. Europe

*Candida* species distribution varies across the European continent. Northern Europe experiences a high contribution from *C. albicans* [54], whilst in central Europe, *C. glabrata* is of increasing prominence [113,114]. Regions of southern Europe consistently report *C. parapsilosis* as the most prevalent NAC strain [98,109,110].

Across northern Europe, *C. albicans* accounts for up to 70% of total IC cases, and *C. glabrata* is the most prevalent NAC species, contributing 10–20% of episodes [39,40,45,53,54,55,56,57]. However, an expected shift towards increasing isolation of more resistant NAC species with increased widespread antifungal use, as seen in the US, has not occurred [39]. A lower disease incidence in the overall population and thus reduced clinical use of antifungals to treat patients with IC may be responsible, limiting the selection pressure posed by such drugs [40]. In Denmark, observations differ and are more akin to the US, where continuing shifts to *C. glabrata* at the expense of *C. albicans* are seen [40,54,59,60]. Data from 2018 show that these species now account for 32.1% and 42.1% of culture confirmed cases, respectively [40]. Higher and combination use of numerous antifungals, notably fluconazole and itraconazole, in Danish healthcare may have driven the observed species disparity with other Nordic countries [54,59,60].

**Figure 4:**
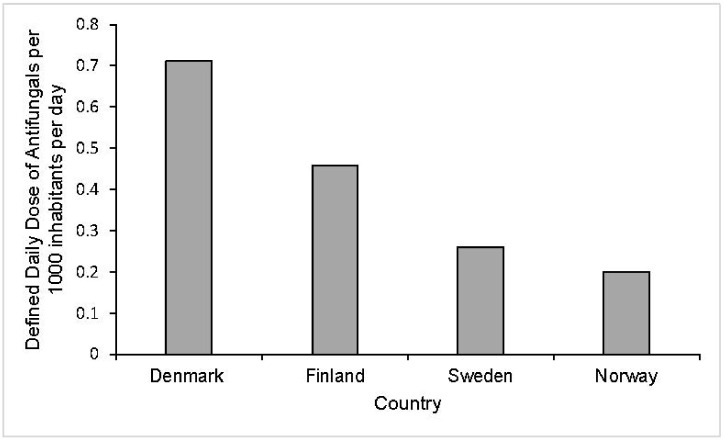
Defined daily dosage (DDD) of antifungal drugs for systemic use per 1000 inhabitants per day in 2011 in healthcare settings across Denmark, Finland, Sweden and Norway. The total use of systemic antifungal drugs is significantly higher across healthcare settings in Denmark than Norway, Sweden and Finland. In Denmark, the DDD per 1000 inhabitants per day was approximately 0.712 compared to 0.459, 0.26 and 0.2 for Finland, Sweden and Norway, respectively [54].

It might be expected that Danish *Candida* isolates would exhibit greater resistance resulting from higher antifungal use and associated selection pressures across Denmark (Figure 4). In fact, the opposite is observed whereby neighbouring countries (e.g., Norway) with lower antifungal use report *Candida* isolates with greater resistance levels, most notably for *C. glabrata* [54]. Explanations for this observation are not available, but it may be due to data contributing to these findings representing a small number of isolates (23 *C. glabrata* isolates from Norway compared to 165 from Denmark); hence, resistance rates calculated from susceptibility testing may not be nationally representative [54,115].

Across central and southern Europe, species and antifungal susceptibility data are comparatively scarce and rely on single/multi-centre studies rather than national programmes. Species distribution trends analogous to the US and Denmark have been reported from institutions across central Europe [113,114,116]. A multi-decade survey by the Fungal Infection Network of Switzerland emphasizes the role of antifungals in these observations, noting that increases in *C. glabrata* isolation and triazole use occurred concomitantly [114]. Studies by others in the region describe a potential reversal of epidemiologic trends, with a marked increase in *C. albicans* and simultaneous decrease in NAC species, driven mostly by reductions in *C. parapsilosis* and *C. tropicalis* prevalence [117,118]. Confirmatory data readouts are required, with important implications if these findings represent ongoing trends. Elsewhere, antifungal use has acted to increase incidence of more resistant NAC species, leading researchers to suggest that recent changes in antifungal practices that favour echinocandin use over triazoles may be implicated [119]. In southern Europe, *C. parapsilosis* is the most common NAC species [98,109,110], with fluconazole-resistant *C. parapsilosis* increasing in prevalence and responsible for a considerably higher neonatal candidemia incidence in the region [92,94,109]. Additionally, *C. parapsilosis* nosocomial transmission is common [97], and therefore outbreaks in an endemic situation cannot be ruled out and may contribute to its increasing isolation further [98]. Overall, given the increasing use of echinocandins in place of triazoles as first-line treatment for IC across Europe, potential changes in species distributions and associated echinocandin resistance should be monitored closely.

#### 5.2.3. South America

In South America, *Candida* species distribution is characterised by high proportions of *C. parapsilosis, C. tropicalis and C. albicans*, contributing >80% of the total IC caseload [111]. *C. parapsilosis* is the predominant NAC species and accounts for ~26.5% of cases across the continent, comparable to observations from southern Europe [109,110,111]. In addition, data from certain countries (Colombia and Venezuela) suggest that *C. parapsilosis* might now be the most common species implicated in infection, surpassing *C. albicans* as the primary causative agent [111]. This may be explained as *C. parapsilosis* is isolated across all age strata whilst on other continents its frequency is heavily concentrated in infant candidiasis.

*C. glabrata*, of major concern in the US and Europe, accounts for just 6% of IC cases across the South American continent. Additionally, low levels of overall antifungal resistance are seen, and it is thought that lower antifungal use might be implicated in both these trends [111,120,121]. *C. glabrata* is of greater prominence in Brazil, increasing in prevalence and currently accounting for 10% of disease events. Interestingly, differences in antifungal (particularly fluconazole) use were found to be negligible in this increase, with defined daily doses (DDD) consistent with those in neighbouring countries. Therefore, it is suggested that an ageing Brazilian population might be the cause, with increasing age a pre-disposing risk for *C. glabrata* infection specifically [111]. This has important implications, because as other South American countries develop an ageing population in the future, they may expect to see increasing *C. glabrata* isolation with inherent resistance. In fact, more recent data from Peru support these claims with *C. glabrata* now approaching 10% of cases there also [120]. Of note, *C. guilliermondii* was found to have a higher incidence than both *C. glabrata* and *C. krusei*, driven by an exceptionally high prevalence in Honduras, accounting for 28% of candidemia cases. High prevalence of *C. parapsilosis* overall and *C. guilliermondii* in specific regions warrants important considerations for antifungal stewardship in South America, as these species contain naturally occurring polymorphisms that increase the likelihood of emerging echinocandin resistance [75,76].

At present, triazoles are still recommended as the first-line therapy for IC. With potential for future increasing isolation of *C. glabrata* and associated triazole resistance, as seen in Brazil, this may change. If this trend continues, treatment guidelines may increasingly recommend echinocandin use over triazoles. In this scenario, additional surveillance will be required to promptly identify trends that may arise in *C*. *parapsilosis* and *C. guilliermondii* echinocandin resistance specifically.

#### 5.2.4. Asia

Across the Asian continent, few distinctive trends in current species distribution and antifungal susceptibility can be concluded due to limited, contrasting data from mostly single-institution retrospective surveillance studies. Generally, *C. tropicalis* might be the primary etiologic agent of IC across west Asia (e.g., Pakistan, India) whilst in east Asia (e.g., China), *C. albicans* remains the most prevalent species with widely varied contributions from NAC species [103,104,105,106,107,108]. This is unsurprising given that China covers a land area of 9.38 million km^2^ and has a population of nearly 1.5 billion, which will inevitably show regional variations in pre-disposing population dynamics and risk factors that influence species distribution.

## 6. Diagnostics

Invasive candidiasis encompasses two distinct disease entities, candidemia BSI and deep-seated tissue candidiasis, with their distinction having important implications for diagnosis [9]. Diagnostics will be considered and split separately here into culture and non-culture-based methods, with blood culture representing the primary diagnostic choice to inform clinicians when IC infection is suspected [122,123]. Increasing development, availability and use of non-culture biomarker tests will likely complement rather than replace culture methods in the future, with combined use promising a new paradigm in patient care and disease management [10,123,124,125,126,127].

### 6.1. Culture-Based Diagnostics

Culture-based diagnostics, involving the detection and growth of viable *Candida* cells predominantly from blood, has been the primary diagnostic tool for decades [122,123]. Culture accurately diagnoses the majority of active candidemia BSI cases, with non-culture diagnostics unlikely to offer significantly lower thresholds of detection [128]. However, ~50% of total IC infection episodes are thought to go undiagnosed by blood culture, reflecting insufficient or absent viable *Candida* cells in circulation for detection [10,83]. These missed diagnoses are largely due to low detection rates and false-negative results for deep-seated tissue candidiasis [9,126,129], resulting from intermittent release of cells from infected tissue sites into circulation or deep-seated candidiasis that is independent of blood-borne candidemia [9,10,126,130]. Sensitivity is also influenced by *Candida* spp., mode of infection and antifungal drugs with *C. glabrata*-implicated candidemia, infection stemming from extravascular sources and use of antifungals at the time of blood draw associated with lower burdens of the pathogen and decreased likelihood of positive culture [83,131,132]. In addition to sub-optimal sensitivity for deep-seated infection and non-active candidemia, blood cultures are associated with highly variable and slow turnaround times, taking up to 8 days until positive culture [83,130]. Sub-optimal sensitivity and slow turnaround times mean that blood culture has limited utility as a definitive diagnostic, with clinicians usually utilising culture for confirmatory purposes and often taking account of multiple suggestive clinical findings to inform clinical decision making instead.

### 6.2. CHROMagar for Species Identification

Widely used mediums for the isolation and growth of *Candida*, such as Sabouraud dextrose agar (SDA) and potato dextrose agar (PDA), are unable to differentiate between *Candida* spp. commonly implicated in IC [133,134]. CHROMagar *Candida* offers a solution, a selective and differential chromogenic isolation medium allowing for presumptive identification of some *Candida* strains of clinical importance through observations of contrasting colony morphology and colour [134,135,136,137]. Contrasting colony colours result from reactions of species-specific enzymes with a proprietary chromogenic substrate [134]. Studies indicate that *C. albicans, C. tropicalis, C. krusei* [135,138] and sometimes *C. glabrata* [135,139] can be differentiated based on these characteristics when grown on this chromogenic medium. Of note, *C. parapsilosis*, due to a wide range of colony colours and morphologies, cannot be distinguished using CHROMagar [140].

The use of CHROMagar medium to identify *Candida* strains implicated in infection, particularly NAC species, can assist clinicians in selecting appropriate antifungal drugs that will be effective and thus may yield significant patient benefit. However, in settings where *C. parapsilosis* is the predominant NAC species, such as in South America and Southern Europe, the utility of CHROMagar will be more limited.

### 6.3. Disease Management and Patient Care Impacts

Culture-based diagnostics have important implications for the patient care and management of IC in healthcare settings, resulting from their poor sensitivity and slow turnaround times that lead to limited clinical utility and gaps in our understanding of the clinical disease spectrum [9,10,122]. Resulting delayed or missed diagnosis of these infections are therefore common and may negatively influence patient prognosis by hindering the initiation of treatment [11]. To mitigate this, current treatment guidelines recommend that early empirical and prophylactic therapy be initiated in high-risk individuals in the absence of an active infection or prior to culture diagnosis [12,13]. Although some individuals will benefit from this practice, its implementation across whole populations of high-risk patients leads to unnecessary use of precious antifungals in many instances, with important implications [12,141]. Of primary concern are the risks of emerging antifungal-resistant strains, as outlined previously, resulting from the high selection pressures caused by widespread, high levels of antifungal use. This may decrease drug efficacy in an already limited number of licensed antifungal agents for IC treatment. Furthermore, the significant healthcare costs implicated in high antifungal use as well as severe side effects endured by recipients are also important [15,72,91]. Antifungal toxicities have both direct and indirect effects on patient health [15]. Indirect impacts may relate to patients’ underlying conditions, with waning compliance to oral medication regimens due to antifungal-induced nausea and vomiting of particular concern among paediatrics [142].

Poor sensitivity of culture-based diagnostics for IC and slow turnaround times have ultimately meant that clinicians must balance the benefits of early empirical or prophylactic therapy in selected high-risk individuals with the risks posed by an increased propensity for emerging antifungal resistance, severe side effects and substantial healthcare costs.

## 7. Future Directions

### 7.1. Non-Culture-Based Diagnostics

Non-culture-based diagnostics or biomarker tests do not rely upon the detection and culture of viable *Candida* cells. As such, they are generally characterised by superior sensitivity and quicker turnaround times, although their clinical use in patient care is still undefined and highly limited [9]. Biomarker tests for IC typically depend upon the detection of *Candida* cell or cell wall components such as DNA, β-D-glucan (BDG) or mannan using PCR, colorimetric tests and immunoassays, respectively, or via host immune responses such as with anti-mannan and *C. albicans* germ tube antibody immunoassays [124,143,144,145]. The T2 *Candida* platform, approved by the US Food and Drug Administration (FDA) in 2014, represents such a test for detecting *Candida* DNA via cell lysis, DNA amplification using PCR and T2 magnetic resonance measurements [146,147]. In addition to superior turnaround times and sensitivity over culture-based methods, the presence of antifungals has been shown to have a negligible impact on T2 *Candida* test performance [132]. The high cost of the T2 *Candida* test may go some way as to explaining why its clinical use in patient care remains relatively limited. As for antibody assays, an important consideration is the immune status of patients, with immunocompromised individuals potentially unable to mount a strong antibody response, which could negatively impact test sensitivity [123,148]. For BDG assays, differentiation between fungi species is not possible given that BDG is a conserved cell wall component of most pathogenic fungi other than the Mucorales and *Cryptococcus*, which lack this carbohydrate in their cell walls [149]. Although these are important considerations, all non-culture-based diagnostics have potentially important uses in future disease management and may ultimately have their greatest impact when used in combination [146,150], increasing the overall probability of promptly diagnosing an IC disease event.

### 7.2. Diagnosis of Deep-Seated Tissue Candidiasis

An advantage of non-culture-based biomarker tests over culture-based methods may be the increased likelihood of detecting deep-seated tissue IC, although some events will remain undiagnosed [141]. This is evidenced by Nguyen et al. [128], where the combined use of PCR and BDG tests identified deep-seated candidiasis in 60% of patients deemed negative for tissue infections based on blood cultures alone [128]. In the same study, PCR biomarker tests elicited superior sensitivity for deep-seated IC over other non-culture-based diagnostics. However, the heterogeneity of available assays means that such findings must be approached with caution and may not be widely applicable [126].

Detecting a larger proportion of deep-seated infections will allow for earlier initiation of antifungal therapy in such instances, bringing about improvements in patient prognosis. This will also expand our understanding of the clinical disease spectrum, mapping IC epidemiology more accurately and providing further insights into *Candida* pathogenesis [9,124]. This includes better defining relationships between the two disease entities, such as the roles of hematogenous seeding in initiating deep-seated infection and the release of *Candida* cells from infected tissue into circulation [125].

However, the clinical utility of non-culture-based biomarker tests as definite diagnostics for IC is restrained by variable positive predictive values (PPVs), referring to the proportion of positive tests that are true-positive results [9,143]. The PPV is heavily dependent upon the pre-test probability of infection, and therefore disease prevalence, in the patient population being tested [126,143,151]. As a result, the use of current biomarker tests as diagnostics will likely be limited to patient populations where the pre-test probability of deep-seated IC is particularly high, such as in ICU patients with mucosal–cutaneous barrier disruption [126].

Combined use of several rapid biomarker tests for *Candida* infections may help broaden our understanding of the IC clinical spectrum by diagnosing a higher proportion of IC disease events, particularly deep-seated infections that were previously missed by conventional blood cultures. However, the utility of these tests as definitive diagnostics is poorly defined and may be limited to specific patient populations with a particularly high pre-test probability of infection.

### 7.3. Biomarker Tests as Prognostic Indicators of Infection

The uncertain performance of biomarker tests as diagnostics means that their precise roles in the management of IC are currently poorly defined [141]. It has therefore been proposed that such tests be used as adjunct prognostic indicators of *Candida* infections rather than providing a definitive diagnosis, with clinicians using other microbiological tests such as culture, and patient risk factors, to inform decision making [126,141]. Biomarker tests may therefore have an important role to play in guiding earlier empirical and prophylactic antifungal treatment [126,141,152]. The utility of biomarkers may also be expanded to antifungal discontinuation in high-risk individuals previously initiated on prophylactic regimens. This is due to high negative predictive values of up to 99% for these tests, meaning that up to 99% of negative tests are true-negative results, allowing clinicians to rule out infection [127,153]. In practice, the feasibility of using biomarker tests to cease antifungal treatment will be determined by the physician. If such an approach is adopted, it has the potential to prevent high-risk patients from receiving drugs that they would not benefit from, restricting antifungal usage where pre-emptive criteria are not met [9]. The use of biomarker tests as outlined has important implications for patient care and IC management, providing an opportunity for reduced unnecessary use of antifungal agents without compromising patient care and individual IC risk. It is hoped that this will allow for a marked reduction in the selection pressure posed by antifungal agents, which is thought to be a key driver in emerging resistance among some clinically important *Candida* spp. Furthermore, biomarker-assisted treatment will reduce the burden of antifungal-induced toxicities by limiting drug use in identified patients who do not require preventative treatment. However, a practical limitation of these biomarker-driven strategies will be the requirement for regular testing of high-risk patients to monitor IC risk over time, something that would be of considerable cost to the healthcare service [152].

The future combined use of blood culture and biomarker tests represents a potential new era in mapping *Candida* diseases in different patient groups by enabling earlier and more accurate detection of blood-borne candidemia and deep-seated candidiasis, as well as transforming patient care and disease management through improved antifungal stewardship [126,127].

### 7.4. Novel Antifungal Targets

In addition to improved antifungal stewardship aimed at preserving the utility of antifungals through the combined use of blood culture and biomarker tests, there is precedence for new antifungal drugs given the paucity of currently available treatment options [15,74]. Novel antifungals that target *Candida* pathogenesis mechanisms, such as hyphal morphogenesis and biofilm formation, are of particular interest compared to current agents that target cell viability via fungistatic or fungicidal activity [74,154,155]. By targeting pathogenesis rather than cell viability itself, future antifungals adopting this framework may benefit from reduced toxicities and subsequent improvements in therapeutic index as well as decreased risks of drug resistance [155]. As described previously, these are some of the main limiting factors associated with current treatments. These antifungals will likely have a narrower spectrum of activity than current broad-spectrum drugs due to their species-specific target of pathogenesis mechanisms. Clinical utility will therefore rely heavily on accurate diagnosis of IC, an area continuing to rely predominantly on the use of slow, insensitive culture-based systems [156,157]. However, increasing development and use of biomarker tests that offer faster, more sensitive disease diagnosis and indications may help realise the potential of such novel antifungals. To date, research has focused on preclinical studies with several compounds showing strong efficacy and promise in vivo in mouse challenge models [156,158,159]. Therefore, although these novel antifungals hold promise, it is likely to be some time before any such agent is available for use in the clinic.

## 8. Conclusions

Invasive candidiasis (IC) is the most common IFD worldwide and represents a continuing public health problem. With shifting patterns of epidemiology largely resulting from healthcare activities, changes in disease management and patient care are urgently needed.

Elucidated from population-based surveillance, increases in overall disease incidence are well-established as the population of immunocompromised individuals susceptible to life-threatening infection has expanded. However, recent data do suggest that in certain settings this trend may be reversing, with improvements in patient risk factor management implicated in these findings. As disease incidence has generally increased, so too have the requirement and clinical use of appropriate antifungal treatments. This has been exacerbated by the use of slow, insensitive culture-based diagnostics, meaning treatment guidelines often recommend empirical or prophylactic strategies for high-risk patients in the absence of proven infection. Many patients therefore receive unnecessary antifungals, with important consequences for their care and disease management more widely, such as severe toxicities and increased risks of drug resistance in an already limited antifungal arsenal. Widespread overuse of antifungals through non-specific preventative strategies is one of the key drivers in *Candida* spp. distribution shifts towards more resistant strains [112,114,160,161]. In particular, an increasing contribution from non-*albicans Candida* spp. at the expense of *C. albicans* is observed globally.

Ever-increasing susceptible populations for opportunistic IC infection and global epidemiologic shifts towards species with greater drug resistance mean that changes in disease management and patient care to preserve and expand the utility of antifungals are of paramount importance. Decreasing unnecessary use of antifungals and their associated selective pressures is an important factor in this, achievable through the use of biomarker tests that allow clinicians to promptly identify and rule out infection. Combined use of biomarker tests and current culture-based diagnostics may therefore yield transformations in disease management and patient care through improved antifungal stewardship. Furthermore, the development of novel antifungals with improved characteristics, targeting *Candida* pathogenesis mechanisms over cell viability, represents a longer-term strategy to expand the repertoire of treatment options beyond the limited number licensed today.

## Figures and Tables

**Table 1:** Overall candidemia population-based incidence rates expressed as the number of cases per 100,000 population across 4 metropolitan sites in four US states from 1992 to 2016.

Year	State Candidemia Incidence (Cases/100,000 Population)			
	Georgia	Maryland	Oregon	Tennessee
1992–1993	9.1	N/A	N/A	N/A
1998–2000	N/A	24.2	N/A	N/A
2008–2011	13.3	26.2	N/A	N/A
2013	9.5	14.4	3.0	8.0
2016	7.5	13.0	3.5	12.5

**Table 2:** Current antifungal agents licensed for the treatment of IC are limited to just three drug classes: the polyenes, triazoles and echinocandins [63].

Antifungal Drug Class	Antifungal Agent
Polyene	Amphotericin B
Triazole	Fluconazole Voriconazole Itraconazole
Echinocandin	Caspofungin Micafungin Anidulafungin

## Data Availability

This study did not generate any new data.
